# Editorial: Metabolic Plasticity of Cancer

**DOI:** 10.3389/fonc.2020.599723

**Published:** 2020-10-23

**Authors:** Sara Rodríguez-Enríquez, Tuuli Kaambre, Rafael Moreno-Sánchez

**Affiliations:** ^1^Departmento de Bioquímica, Instituto Nacional de Cardiología, Ciudad de México, Mexico; ^2^Chemical Biology Laboratory, National Institute of Chemical Physics and Biophysics, Tallinn, Estonia

**Keywords:** mitochondrial metabolism, glycolysis, cancer stem cells, drug targeting, malignant cancer

The metabolic reprogramming strategies used by cancer cells to survive and proliferate include changes in their energy metabolism status and enzyme machinery. During carcinogenesis, substantial changes in tissue oxygen, glucose (and other carbon sources) and pH occur which affect cellular energy management. Malignant cells show enhanced glycolytic capacity even in the presence of a normal oxygen concentration (Warburg hypothesis). The Warburg hypothesis also states that the mitochondrial function of cancer cells is impaired. The glycolytic part of the Warburg hypothesis has been solidly documented for the great majority of cancer types; in contrast, the mitochondrial part has been matter of intense research and controversy the last 20 years. It should be noted that in addition to OxPhos, other mitochondrial functions are (i) anaplerotic supply of intermediary metabolites for the synthesis of amino acids, fatty acids, cholesterol, glucose, and heme; (ii) ROS generation and oxidative stress management; (iii) apoptosis onset and progression; and (iv) Ca^2+^ homeostasis. These essential mitochondrial functions for cell growth and survival cannot be fulfilled by the glycolytic pathway or any other metabolic pathway.

## Cancer Mitochondrial Metabolism

Recently it has been demonstrated that some typical waste metabolites produced by mammalian cells such as ${\text{NH}}_{4}^{+}$ or lactate, whose metabolic transformation requires functional mitochondria, may support cancer cell growth.

In this regard, the contribution by Prado-García et al., describe that, under a mild lactic acidosis and normoxia, A427 lung cancer cells are able to oxidize exogenous lactate favoring OxPhos and cell growth with no change in glucose consumption but diminished glycolysis, whereas A549 lung cancer cells display diminished glucose consumption, glycolysis, and OxPhos. Transcriptional response of both cancer cell lines is also different. Thus, it seems that lactic acidosis may have multiple regulatory effects on glycolysis and OxPhos. Further studies analyzing separately the effects of simple acidosis (at constant lactate), a wider range of lactate concentrations (at constant pH) and exposure times may help elucidating how energy metabolism is regulated by lactic acidosis in lung cancer cells.

Louie et al., report that MCF-7 breast cancer cells exposed to different combinations of physiological substrates (glucose, glutamine, pyruvate) activates OxPhos, displaying ATP production rates 60% greater than those shown by cells cultured with each individual substrate. This response is not observed in non-transformed C2C12 myoblasts. It would be interesting to detect and characterize this metabolic plasticity in other cancer cell types by using physiological substrate combinations including free fatty acids, lactate and ketone bodies, to establish whether this feature is unique to cancer.

Moreno-Sánchez et al., propose a novel anabolic role for mitochondrial glutamate dehydrogenase (GDH) as a ${\text{NH}}_{4}^{+}$ fixing enzyme for supporting growth of metastatic cancer cells. The kinetic constants experimentally determined in this study clearly indicate that GDH reverse reaction (from 2-oxoglutarate to glutamate and NADPH production) is favored under physiological conditions to sustain cancer growth. However, it remains to be determined why ammonium is not toxic for cancer cells and why metastatic cells are apparently better equipped to use it for cellular functions.

Koit et al. demonstrate, by using a citrate-isotope tracing method and ADP-stimulated O_2_ consumption of permeabilized cells, that aggressive human breast cancer postoperational samples maintain high respiration rate and high mitochondrial citrate efflux compared to less aggressive subtypes. Citrate fluxes and O_2_ consumption of two of the most used breast cancer cell lines, MCF-7 and MDA-MB231, differ from those shown by breast cancer tissue samples. This is expected because cancer cell lines are grown as bi-dimensional cultures, whereas cancer tissue is a tri-dimensional system. This tri-dimensional organization (i.e., cell-matrix interactions, variations in nutrient supply due to glucose, lactate, pH, and oxygen gradients; as well as variations in the expression of transcription factor and oncogenes), leads to changes in gene regulation, enzyme expression/ activity, and metabolic fluxes with respect to monolayer cell cultures and cell suspensions.

Lemos et al., demonstrate the importance of fatty acid β-oxidation in the development of the metastatic phenotype. By using microcalorimetry, they show that metastatic cancer cells release more heat than non-metastatic cells, correlating with an overexpressed uncoupling protein 2 (UCP-2). It should be noted that both β-oxidation and UCP-2 reaction (H^+^ transport) are mitochondrial processes. The carnitine/palmitoyl-CoA transferase-1 inhibitor etomoxir decreases heat release by metastatic cells, indicating that β-oxidation is involved in thermogenesis. In addition to extent these observations to more metastatic cancer cell lines and biopsies, it would be relevant to show that metastatic cells indeed actively oxidize fatty acids by assessing O_2_ consumption rates and protein levels. These observations also indicate that fatty acid β-oxidation and UCP-2 may be anti-cancer targets and that the novel biophysical approach used could be helpful for metastasis detection.

The contribution by Bouchez et al., using yeast cells as a suitable model to study the Warburg effect (where all parameters involved and their modulation can be reconstituted) demonstrates that mitochondria are not dysfunctional and importantly, that OxPhos is required to promote cell growth.

Previous work from Rodriguez-Enriquez group have also shown that OxPhos is the predominant ATP supplier (60–80% under normoxia and normoglycemia), with glycolysis having a minor role, for growth and survival of human cervix HeLa cells and microspheroids, human breast MCF-7 and MDA-MB231 cells, and rodent AS-30D ascites hepatoma cells. Glycolysis becomes the major ATP provider (55–80%) when cancer cells are subjected to severe and prolonged hypoxia (0.1% atmospheric O_2_, 24–48 h) or hypoglycemia (<5 mM glucose). Recent work describes that the non-steroidal anti-inflammatory drugs celecoxib and its analog dimethyl-celecoxib, at nanomolar (3D) or micromolar (2D) concentrations, increase cisplatin, paclitaxel and doxorubicin efficacy (>60%) against human cervix cancer cell growth, OxPhos and invasiveness (>90%), without apparent effect on human HUVEC and fibroblasts (*unpublished data*).

## Cancer Glycolysis

The study performed by Piga et al., in ovarian cancer xenografts shows that some glycolytic markers such as the monocarboxylate transporter MCT4 are negatively associated with survival. In turn, Vlaski-Lafarge et al., demonstrate that normal hematopoietic stem cells develop a cancer stem cell-like phenotype (by over-expressing the canonical markers CD34+ and ALDH) when they are experimentally subjected to anoxic and/or aglycemic stresses. They found that under ischemia-likeconditions, primitive hematopoietic cell mitochondria remain functional for sustaining cellular homeostasis.

Prasad-Yadav et al., review the energy metabolism of CSC derived from different cancer tissues. They conclude that CSC from breast, glioblastoma and osteosarcoma predominantly depend on glycolysis for survival while CSC from glioma, pancreas and lung depend on OxPhos (which is mainly sustained by free fatty acids and ketone bodies oxidation). Thus, the identification of the main ATP supplier may be helpful to design strategies to kill selectively cancer cells by using specific inhibitors targeting energy metabolism.

In the study by Avagliano et al., with metastatic cutaneous melanoma (CM) it is shown that both glycolysis and OxPhos are actively involved in cellular ATP formation, although the main energy supply relies on glycolysis. In addition, it is demonstrated that glycolysis activation in CM is mediated by the BRAF/MAPK signaling pathway containing a BRAF kinase mutant. Consequently, the use of BRAF/MAPK inhibitors induces in MC cells metabolic re-programming from glycolysis to OxPhos.

## Signaling

As described in Avagliano et al. study, metabolic reprogramming in cancer cells is tightly regulated by transcriptional factors, protein kinases and/or oncogenes. Among them, the forkhead box protein C2 (FOXC2), casein kinase (CK2), and adenylate kinase (AK) have been proposed as cancer markers or even targets to deter cancer progression. Hargadon and Williams use RNA-seq dataset to detect novel tumor-promoting functions of FOXC2, including its role as a regulator of mitochondrial morphology and metabolism. Silva-Pavez and Tapia compile evidence from literature suggesting that CK2 is a switch modulator of the mitochondrial function in a PTEN-dependent mode. They conclude that PTEN, as CK2 substrate in the PI3K/Akt signaling pathway, can regulate several downstream key targets like HIF-1α and mitophagy. Therefore, the specific inhibition of CK2 in cancer cells may be a potential therapeutic strategy against cancer development.

Klepinin et al., propose AK (adenylate kinase, an ubiquitous and highly efficient enzyme involved in the adenylate nucleotide cellular homeostasis) as a novel cancer therapeutic target. They demonstrate that different AK isoforms are used by cancer cells for rewiring energy metabolism to support tumor progression and metastasis. These observations allow concluding that although cancer cells maintain a high glycolytic rate, the main ATP production derive from OxPhos. Thus, anti-mitochondrial drug therapy combined with signaling targets (FOXC2, CK2, or AK) may be an adequate adjuvant strategy to arrest proliferation of OxPhos-dependent neoplasias.

It is convenient to emphasize that most of the studies published in the present special issue of *Frontiers in Oncology, Cancer Metabolism section* have focused on directly assessing the metabolic function (i.e., cell growth, O_2_ consumption, glucose consumption, lactate production, enzyme activities). This takes relevance because, to date, many studies published in high impact journals only rely on determining mRNA or protein levels to make inferences on the functional consequences of using for instance inhibitors or genetic manipulations. As cancer cells can only depend on both glycolysis and OxPhos for ATP supply, like non-cancer cells, the challenge appears to be in designing effective drugs that better and more specifically target cancer cells and, in particular, their mitochondria, which are now postulated as essential in all cancers.

## Author Contributions

All authors listed have made a substantial, direct and intellectual contribution to the work, and approved it for publication.

## Conflict of Interest

The authors declare that the research was conducted in the absence of any commercial or financial relationships that could be construed as a potential conflict of interest.

